# Predictive validity of three home fall hazard assessment tools for older adults in Thailand

**DOI:** 10.1371/journal.pone.0244729

**Published:** 2020-12-31

**Authors:** Charupa Lektip, Sarawut Lapmanee, Thanapoom Rattananupong, Vitool Lohsoonthorn, Arnond Vorayingyong, Thira Woratanarat, Kitti-On Sirisuk, Plaiwan Suttanon, Rewwadee Petsirasan, Parinya Kitidumrongsuk, Wiroj Jiamjarasrangsi

**Affiliations:** 1 Department of Physical Therapy, School of Allied Health Sciences, Walailak University, Nakhon Si Thammarat, Thailand; 2 Preclinical Department, Faculty of Medicine, Siam University, Bangkok, Thailand; 3 Department of Preventive and Social Medicine, Faculty of Medicine, Chulalongkorn University, Bangkok, Thailand; 4 Department of Interior Architecture, Faculty of Architecture, Chulalongkorn University, Bangkok, Thailand; 5 Department of Physical Therapy, Faculty of Allied Health Sciences, Thammasat University, Bangkok, Thailand; 6 School of Nursing, Walailak University, Nakhon Si Thammarat, Thailand; Chiang Mai University Faculty of Medicine, THAILAND

## Abstract

This study aimed to examine the predictive validity of two internationally well-known instruments, the Modified Home Falls and Accidents Screening Tool (Modified HOME FAST) and the Modified Home Falls and Accidents Screening Tool-Self Report (Modified HOME FAST-SR), and the newly developed Thai Home Falls Hazard Assessment Tool (Thai-HFHAT) (69 items) in predicting falls among older Thai adults. It also aimed to examine the predictive validity of the two abbreviated versions (44 and 27 items) of the Thai-HFHAT, which were developed post hoc to accommodate older adults’ limited literacy and poor vision and to facilitate the identification of high-impact home fall hazards that are prevalent in the Thailand context. A prospective cohort study was conducted among 450 participants aged 60 years and above who were assessed by the aforementioned tools at baseline, for which data on fall incidence were then collected during the one-year follow-up. The Cox proportional hazard model was applied to estimate hazard ratios (HRs); then, Harrell’s C-statistics and receiver operating characteristic (ROC) analyses were conducted to identify the best cutoff point, sensitivity and specificity for each instrument. The results showed that the fall hazard rate was 2.04 times per 1,000 person-days. Taking into account both the predictive validity and applicability, the Thai-HFHAT (44 items) was found to be the most suitable screening tool due to its highest sensitivity and specificity (93% and 72%) at the cutoff score of 18. In conclusion, our study showed that these internationally validated home fall hazard assessment tools were quite applicable for Thailand, but further tailoring the tools into a specific local context yielded even more highly valid tools in predicting fall risk among older Thai adults. Although these findings were well reproducible by inferring from the internal validation results, further external validation in the independent population is necessary.

## Introduction

Falls are the second leading cause of unintentional injury-related death worldwide, with the highest death rates being among adults over the age of 65 years. Over 80% of fall-related deaths occur in developing countries, with the Western Pacific region and Southeast Asia accounting for 60% of these deaths [1]. In Thailand, the six-month prevalence of falls among older Thai adults in 2016–2017 ranged between 16.9–18.5%, with a corresponding fall-related death rate of 50 per 100,000 older adults [2, 3]. Among these, 34.5–47.5% took place inside the home, with a six-month prevalence of household falls of 5.8–8.8%. Since environmental hazards are the leading cause of falls, and many falls in older adults occur in the home [4, 5], accurately identifying home fall hazards that require intervention is fundamental for reducing fall risk among this population [6, 7]. However, home environmental fall hazard assessment tools currently used in Thailand and other Southeast Asian countries sharing similar contexts are mostly nonstandardized or generic in nature, which may lead to inaccurate findings [8].

Reliable home fall hazard assessment tools should contain evidence on at least five psychometric properties, including content, construct and criterion validity, interrater reliability, and responsiveness [9]. Among these, predictive validity—one subtype of criterion validity—is the most important type of validity to strengthen the purpose and accuracy of the instrument in the identification of home environmental hazards that significantly contribute to the risk of falls in older adults [10]. Unfortunately, a systematic review by Romli et al. to identify standardized home fall hazard assessment tools and to evaluate their psychometric properties [11] reported that among 19 identified instruments, only four instruments, namely, the Home Falls and Accidents Screening Tool (HOME FAST), the Home Safety Self-Assessment Tool (HSSAT), the In-Home Occupational Performance Evaluation (I-HOPE), and the Westmead Home Safety Assessment (WeHSA), have adequate evidence on predictive validity in relation to fall risk.

Among these five reliable instruments, HOME FAST [9] is the only instrument that has evidence on all these psychometric properties. It was specifically developed for older fallers and focused on person-environment interactions when evaluating home hazards. Its predictive validity is among the highest quality [12]. In addition, it has favorable clinical utility in terms of accessibility (free/open), ease of use (usable by lay health workers), duration (25 items with 20 minutes of administration), and training requirement (self-reading of the manual, but a brief one-hour introductory workshop is desirable) [11] Furthermore, a self-report version, Home Falls and Accidents Screening Tool-Self Report (HOME FAST-SR) (87 items), was also developed, which relied on self-report by the older persons themselves [13]. The HOME FAST-SR had moderate agreement with the traditional HOME FAST and had satisfactory predictive validity over a six-month period [14]. It has been widely adopted at the international level in at least four languages, including English, Persian, Mandarin and Malay [15–18]. However, as environmental factors vary according to geography, culture, and architectural design, international application of this instrument needs a certain level of adaptation to suit the local context [19].

One standardized fall risk assessment tool exists for Thailand specifically, namely, the Thai Falls Risk Assessment Test (Thai-FRAT) [20]. Even though this tool had high sensitivity (92%) and specificity (83%), it relied mainly on the person’s internal factors with only one question about home environmental factors. Therefore, we developed the Thai Home Falls Hazard Assessment Tool (Thai-HFHAT) to suit the geography, culture, and architectural design of Thailand. This tool had 69 items and was constructed based on the traditional HOME FAST and HOME FAST-SR, other internationally published instruments [21–25], as well as input from local literature [5, 26, 27], expert opinion, Thai elderly persons, and caregivers. Except for predictive validity—which has not yet been assessed—its other psychometric properties were excellent and included item- and scale-level content validity indices (I-CVI and S-CVI) of 0.7 and 0.9, an intraclass correlation (ICC) representing an interrater reliability of 0.87 (95% confidence interval or CI: 0.78–0.93), and an ICC representing the 2-week test-retest reliability of 0.78 (95% CI: 0.58–0.89) [28].

This study aimed to examine the predictive validity of two internationally well-known instruments: the Modified Home Falls and Accidents Screening Tool (Modified HOME FAST) and the Modified Home Falls and Accidents Screening Tool-Self Report (Modified HOME FAST-SR), and the newly developed Thai Home Falls Hazard Assessment Tool (Thai-HFHAT) (69 items). The Thai-HFHAT (69 items), with its large item number to cover the possible whole range of home fall hazards, however, may be too burdensome for older adults with limited literacy and visual problems, which are prevalent in Thailand. Therefore, its two abbreviated versions (44 and 27 items) were created post hoc to accommodate this problem. Due to the proper procedure for item selection, these two abbreviated versions of Thai-HFHAT will also facilitate medical personnel to better focus on the identification and management of more limited numbers of home fall hazards but with high impact on the future falls of older adults that are frequently encountered in the Thailand context. Specifically, the objective of this study was to determine the area under the receiver operating characteristic curve (AUC), sensitivity and specificity of the Modified HOME FAST, Modified HOME FAST-SR, Thai-HFHAT (69 items) and its two abbreviated versions (44 and 27 items) in predicting fall events during a one-year follow-up among Thai adults ≥ 60 years old residing in a district in southern Thailand.

## Materials and methods

### Study subject

This prospective cohort study was conducted in Tha Sala District, Nakhon Si Thammarat Province, southern Thailand. The study was approved by the Research Ethics Review Committee, Faculty of Medicine, Chulalongkorn University (IRB reference no. 492/61) before being conducted. In addition, the study participants read the "Explanation Fact Sheet for Participants" and signed the "Submission Agreement for Volunteers" before starting the study.

**Fig 1:**
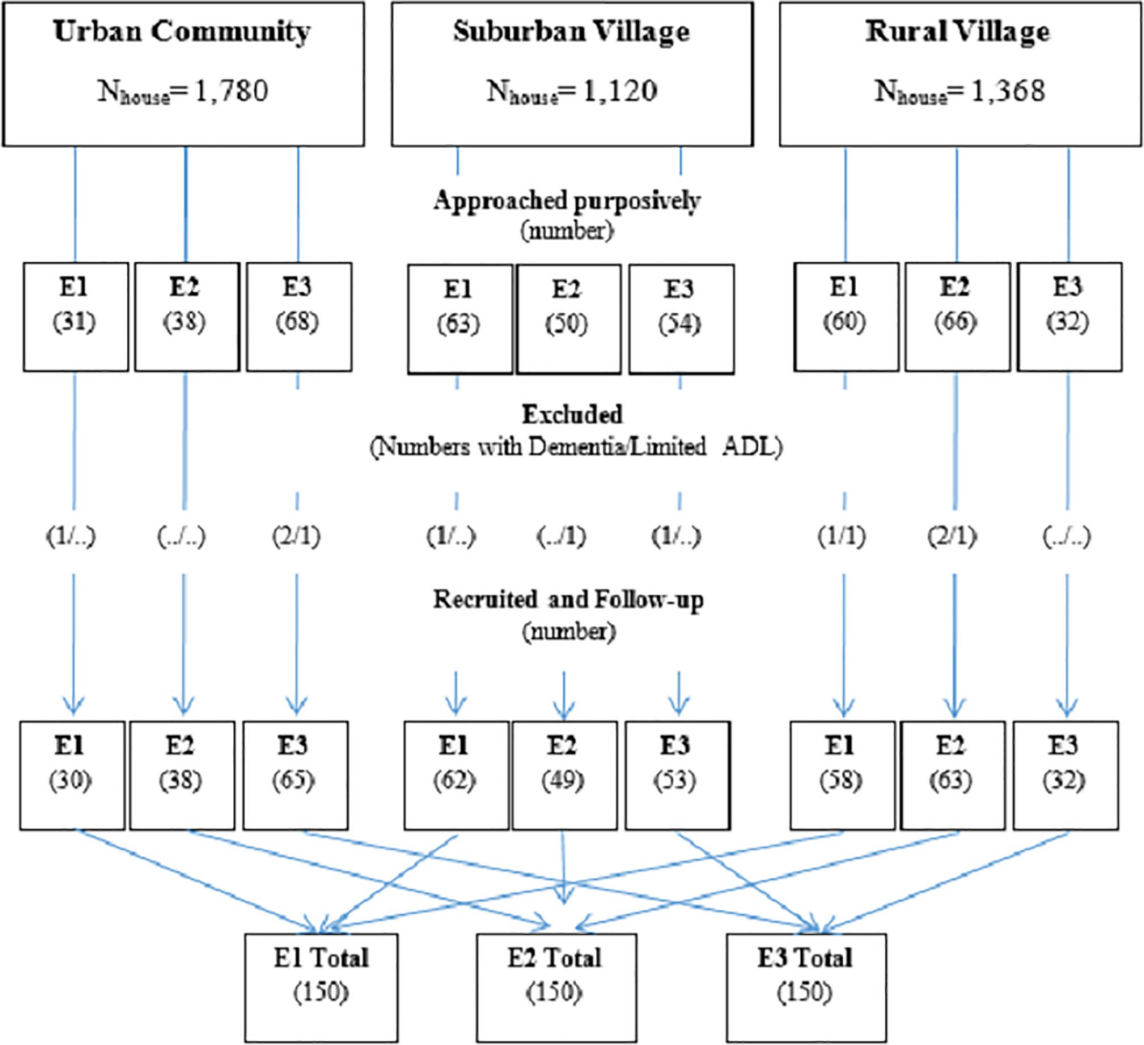
Flow chart of participant recruitment.

The target population included those aged ≥ 60 years who resided in the study area and were fluent in Thai. Those who could not perform activities of daily living and those with dementia were excluded. The activities of daily living were assessed by the Barthel Activities of Daily Living Index (with scores of ≤ 4 out of a total score of 20 considered to be total dependency and thus excluded), while dementia was screened by the Mini Mental State Examination in the Thai version (MMSE-Thai) 2002 (with scores of ≤ 14 out of a total score of 23 for participants without any education; ≤ 17 and ≤ 22 out of a total score of 23 for those with a 7^th^ grade or lower and those with an 8^th^ grade or higher education, respectively, were considered cognitively impaired and thus excluded). Sample size calculation was based on the following formula [29]: n_control_ = (Z ^2^_α/2_ P (1-P)/d ^2^ and n_total_ = n_control_/(1-prevalence), where n_control_ = number of nonfallers, n_total_ = number of total subjects, P = expected specificity (0.8) [20], d = allowable error (0.05), Z_α/2_ = standard values for type I error at an α level of 0.05 (1.96), and prevalence = prevalence of falls among Thai elderly individuals (0.27) [2]. Considering a possible drop-out rate of 25%, the required sample size was 450 participants. To be able to represent all main types of houses in Thailand, quota sampling was utilized, where 150 participants each were recruited from each of three types of houses, namely, a one-story elevated house, a one-story non-elevated house, and a two- or more-story house [3]. Since no sampling frame was available for randomly selecting participants according to the types of houses, we conducted field visits to three residential communities/villages (urban, suburban, and rural, where main types of houses differed) and purposively recruited eligible and consented participants serially until the required numbers of participants in each type of house were met (Fig 1).

### Data collection

#### Demographics and health history

Demographic characteristics of participants, including age, sex, marital status, house type, visual acuity, balance ability, and history of falling in the previous six months [30], were collected using standardized questionnaires. Visual acuity was assessed using a Snellen chart (“good” being able to read more than half the numbers on the Snellen chart at a distance of 6/12 meters, and “poor” otherwise) [20], while balance ability was assessed with a one-leg balancing test for 10 seconds (“good” being able to stand on one leg for 10 seconds or longer and “poor” otherwise) [31].

### Home fall hazard assessment

Three instruments were used in the home fall hazard assessment, including the Thai versions of the Modified HOME FAST and the Modified HOME FAST-SR and the newly developed Thai-HFHAT. The assessments were conducted at the beginning of the study. The summarized specifications of these instruments are presented in Table 1 (see S1 Table for more detail). In addition, the two abbreviated versions of the Thai-HFHAT (44 items and 27 items) were developed post hoc based on two data-driven methods. The 44-item version was developed based on multivariable Cox regression modeling, while the 27-item version was developed based on the frequent home fall hazards question items identified by the participants and relevant to the Thailand context [5, 32]. Details of these two data-driven methods are provided later in the Statistical Analysis” subsection and S2 Table.

**Table 1:** Detail specification of home fall hazard assessment instruments.

Specification	Modified HOME FAST	Modified HOME FAST-SR	Thai-HFHAT (69 items)	Thai-HFHAT (44 items)	Thai-HFHAT (27 items)
Question item					
flooring	3	9	23	16	13
furniture	1	3	2	1	1
lighting	3	10	8	6	1
bathroom	6	25	10	9	5
storage	1	1	1	1	1
stairways/steps	4	14	18	10	2
mobility	3	9	2	1	1
around the house	2	9	3	1	1
shoes	1	5	1	0	1
pets	1	2	1	0	1
Total	25	87	69	44	27
Score	25	25	69	44	27
Scoring procedure	1 item = 1 point	convert 87 items into 25 points	1 item = 1 point	1 item = 1 point	1 item = 1 point
Assessor	Physical Therapist	Elderly/^†^Caregiver/VHV^†^	Elderly/Caregiver/VHV^†^	Elderly/Caregiver/VHV^†^	Elderly/Caregiver/VHV^†^
Duration [28]	20 Minutes^‡^	60 Minutes^‡^	45 Minutes^‡^	30 Minutes^‡^	20 Minutes^‡^
Sources	Translation and cultural adaptation	Translation and cultural adaptation	Thai and international literature + Focus group + Expert opinion	Multivariable Cox regression modeling	Frequent responses (by ≥80% of participants) on each of 9 question domains¥

VHV = Village health volunteer.

^†^ The elderly was the main self-assessor, caregiver assessed when the elderly was unable to assess by him/herself, and the responsible VHV assessed (without prior training) when the elderly was unable to assess by him/herself and there was no caregiver.

^‡^ Assessment time used for Modified HOME FAST, Modified HOME FAST-SR, and Thai-HFHAT (69 items) was measured during the validation study, while those for Thai-HFHAT (44 items) and Thai-HFHAT (27 items) were measured post hoc.

¥ Developed based on the frequent home fall hazards in the Thailand context and identified by at least 80% of the participants.

Unique features of Thai housing and living styles that differed from those in Western countries were taken into account in the development of home fall hazard assessment instruments. These included the following:

- In the “Flooring” domain, unused cloth is frequently used in place of mats/rugs and floors of different levels.
- In the “Mobility” domain: many older Thai adults sleep on the floor instead of in a bed.
- In the “Bathing” domain, bathtub and shower enclosures are rarely used, and squatting-type toilets are prevalent.
- In the “Around the house” domain, Thai people usually take off their shoes before entering the house, leaving them at the doors and sometimes obstructing the entrance.
- In the “Shoes” domain, Thai people normally walk barefoot inside the house.

Thai versions of HOME FAST and HOME FAST-SR (namely, the Modified HOME FAST and Modified HOME FAST-SR) were developed from the original English versions upon permission, using the standard processes for translation and cultural adaptation [33]. First, the original English versions were independently translated into Thai by two translators with good bilingualism. Second, the principal investigator (CL) and the two translators worked together in comparing the two translated versions and harmonizing their differences in words, terms or expressions and in conducting the necessary cross-cultural adaptation for deriving the conciliation version. Third, the conciliation version was back-translated from Thai to English by another expert translator. Finally, the principal investigator (CL) and all involved translators finalized ideas for consistency between the back translation and the original English version.

Some questions in the original HOME FAST and HOME FAST-SR were omitted due to their irrelevancy in Thai-style houses, including those in the “Flooring” (item number 2), “Furniture” (item number 10), “Mobility” (item numbers 22, 23, 31), “Bathing” (item numbers 40–56), “Around the house” (item number 79), and “Shoes” (item number 89) domains.

The Thai-HFHAT (69 items) was newly constructed by the investigators based on the two aforementioned and other internationally published instruments [21−25] as well as with input from local literature [5, 26, 27], expert opinion, Thai elderly persons and caregivers. This resulted in some differences between the Thai-HFHAT and the original HOME FAST-SR. In addition to omitting some question items as described in the previous paragraph, further modifications were also made to generate the Modified HOME FAST-SR.

First, for simplicity, the following groups of question items in the original HOME FAST-SR were collapsed into one question item: item numbers 63, 66 and 67, 68 and 69, 72 and 73, 75 and 76 in “Steps/Stairs”; item numbers 80 and 81 in “Around the house”; item numbers 90–92 in “Shoes”; and item numbers 93–95 in “Pets”.

Second, phrases in item numbers 1, 3, 4, 5 and 8 in “Flooring”, item number 28 in “Mobility”, item number 38 in “Bathing”, and item number 87 in “Around the house” were significantly changed.

Third, item number 27 in “Mobility”, item numbers 57 to 61 in “Bathing”, item numbers 77 and 78 in “Steps/Stairs”, and item number 88 in “Around the house” were added to accommodate the current housing styles.

The remaining 35 question items were still similar to the original instrument. Finally, pictorial illustrations for all 9 domains were also added to facilitate understanding by older adults/caregivers who filled out the instrument (see S1 Table for more detail).

Detailed procedures for developing these three instruments as well as their test results for inter-instrument agreement, content validity, and interrater and testretest reliability were published previously [28]. Time durations used in the assessment by Modified HOME FAST, Modified HOME FAST-SR, and Thai-HFHAT (69 items) were measured during the validation study and were 20, 60, and 45 minutes; those for Thai-HFHAT (44 items) and Thai-HFHAT (27 items) were measured post hoc and were 30 and 20 minutes, respectively.

Demographic and health history data collections and home environmental fall hazard assessments were conducted at baseline by the principal investigator (CL), who is a physical therapist, two undergraduate physical therapy students with one hour of training, the participant, his/her caregiver, and a village health volunteer (VHV). The Modified HOME FAST was filled out by the principal investigator and two physical therapy students, while the Modified HOME FAST-SR and Thai-HFHAT were filled out by the participant, his/her caregiver if the participant was illiterate or unable to fill out the forms for any reason, or the responsible VHV for the participant with such conditions. Home fall hazard assessments by the principal investigator/physical therapy students and the participant/caregiver/VHV were conducted simultaneously at the participant’s home on the same day without consulting each other. Data about living and working behavior in the home were collected by the principal investigator interviewing the participant. The participant/caregiver/VHV was allowed a five-minute break between the assessments with the Modified HOME FAST-SR and the Thai-HFHAT. Missing data were checked and completed before leaving each home.

### Fall outcome assessment

Falls were defined as accidentally changing body positions in relation to the ground. It does not include falling down to rest on furniture, walls, or other objects inside the home and around the home of the elderly and excluded falls from being hit by a person or crashing [34]. Falls were treated as repeated outcomes, that is, all fall event(s) occurring during the follow-up period for each individual participant were taken into account. Data about the consequence of the fall were also collected and included abrasions, contusions, open wounds, rib fractures, femoral fractures, wrist fractures, head injuries, and other injuries, or no injury at all.

Falls were assessed during the twelve-month follow-up period from October 2018 to October 2019. After the baseline home fall hazard assessment, each participant received a “fall calendar” for recording fall occurrence(s) during the follow-up period. The principal investigator (CL) then called each participant monthly to collect the data about fall occurrence, including the date and nature of the fall, environmental context, severity and treatment needed.

### Statistical analysis

Characteristics of the study subjects were analyzed. The frequency with percentage for categorical variables and the mean with standard deviation (SD) for continuous variables are presented. Chi-square (or Fisher’s exact test when more than 20% of cells had expected frequencies < 5) and independent *t*-tests were used for categorical and continuous variables, respectively, to compare fallers and non-fallers.

Interesting assessment tools in this validity study included the Modified HOME FAST, Modified HOME FAST-SR, Thai-HFHAT (69 items) and its two abbreviated versions (44 and 27 items), which were developed post hoc. In the development of the abbreviated Thai-HFHAT (44 items), the Cox proportional hazards model with conditional risk set (Prentice, Williams & Peterson: PWP-ET) model for determining the association of the Thai-HFHAT (69 items) and fall risk was conducted by treating its 69 question items as the independent variables instead of its total score. The hazard ratio (HR) and the corresponding 95% confidence interval (95% CI) were utilized as measures of association. Univariate analysis was initially conducted to determine the hazard ratio (HR) of each item in the association with fall occurrence. Question items that were related to fall occurrence with a *p*-value of < 0.25 were candidates for further statistical modeling [35]. Later, in the statistical modeling, a stepwise selection procedure was conducted [36], leaving only question items with *p* < 0.10 in the final model. Potential confounding effects of internal factors such as sex, age, visual acuity, body balance ability, history of falls, and stroke were taken into account in this statistical model. This resulted in the first abbreviated version of the Thai-HFHAT, with 44 question items (S2 Table). For the development of the abbreviated Thai-HFHAT (27 items), question items about home fall hazards in the abbreviated Thai-HFHAT (44 items) that were frequently reported in the Thailand context [5, 32] and identified by at least 80% of the participants were selected. These items included 10 items for flooring, 3 items for furniture, 1 item for lighting, 7 items for bathrooms, 1 item for storage, 2 items for stairways/steps, 1 item for mobility, 1 item for shoes and 1 item for animals, resulting in 27 items remaining in this abbreviated version (S2 Table).

**Fig 2:**
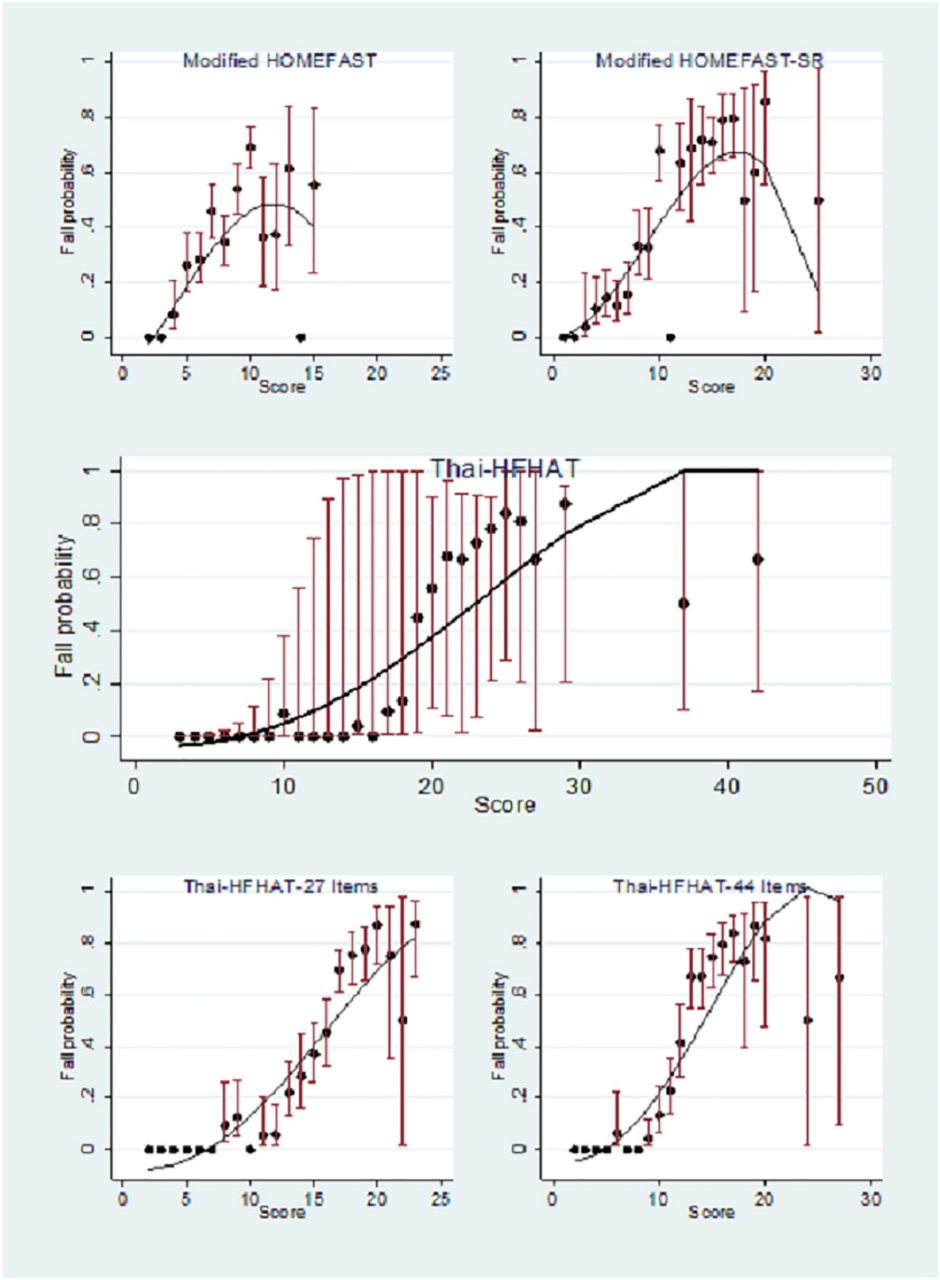
Observed and predicted fall probability of the home fall hazard assessment scores.

Predictive validity analysis began by examining the relationship pattern between the home fall hazard assessment scores and fall probability by incorporating the fractional polynomial (FP) method into the Cox analysis [37]. Detailed results are shown in S1 File. Model performance, including both calibration and discrimination, was then evaluated. In the model calibration, the assessment score-fall probability curves derived using the FP method were estimated and compared with the observed fall probability (Fig 2). The calibration checking procedure suggested by Royston was also conducted [38] (S2 File). The intercept of the calibration plot should be near zero, while the slope of the calibration plot should be 1, which reflects a perfectly calibrated model.

Discrimination was assessed by Harrell’s C-statistics [39] and the receiver operating characteristic (ROC) curve. The corresponding sensitivity, specificity, and positive and negative predictive values for the specific cutoff point were then determined for each tool [35]. The interpretation of the area under the ROC curve (AUC) could be stated as follows: 0.5 = no discrimination, 0.7 to 0.8 = acceptable, 0.8 to 0.9 = excellent, and more than 0.9 = outstanding [40]. The optimal cutoff score for each assessment tool was determined by Youden's J statistic [36]. The relative predictive powers among the tools were compared by using out-of-sample predictions based on C-statistics [41, 42].

The potential for overfitting and optimism of the prediction model [43, 44] was managed by internal validation. By this procedure, bootstrap-corrected Harrell’s C-statistics were performed using 1,000 bootstrap resampling cycles. The difference between the AUC estimated from using the original data and the AUC estimated from the bootstrap resample was considered a measure of optimism. The bootstrap optimism-corrected AUC or C-statistics were computed by subtracting the optimism from the original AUC to estimate an unbiased measure of the abilities of predictive models to discriminate among the elderly individuals in the development cohort with respect to their falls [44]. In addition, the discriminative performance of the dichotomized home fall hazard scores was further examined in these original data by categorizing subjects into “Low” and “High” score groups based on the previously identified optimal cutoff for each home fall hazard assessment tool and then determining the future fall hazard ratio by treating the “Low” score group as the reference. The observed fall probabilities in the two groups were also examined. Last, the agreement on differentiating the participants into “Low” and “High” fall hazard groups among five assessment tools was examined by Fleiss’ and Cohen’s kappa, respectively, for overall and pairwise agreements [45]. The level of agreement based on the kappa value was determined as follows: 0.20 = none; 0.21–0.39 = minimal; 0.40–0.59 = weak; 0.60–0.79 = moderate; 0.80–0.90 = strong; and above 0.90 = almost perfect [46].

The statistical significance level was set at 0.05 for all analyses. STATA Version 15 (StataCorp. 2017. Stata Statistical Software: Release 15. College Station, TX: StataCorp LLC) for Windows was used to perform all data analyses.

## Results

### Fall incidence

During the one-year follow-up period, 123 out of 450 older adults reported the occurrence of at least one fall incidence, accounting for a cumulative incidence rate of 27.33% (95% CI: 22.72, 32.61) per year. The corresponding number of incident falls was 334 during the total follow-up period of 163,550 person-days, resulting in a fall incidence density of 2.04 (95% CI: 0.06, 12.38) falls per 1,000 person-days. Among those who fell, the numbers of falls ranged from 1 to 17, with a median of 1. Out of 334 falls, 232 (69.46%) resulted in no injury, 84 (25.15%) contusion, 14 (4.19%) abrasion, and 4 (1.20%) severe injuries, such as severe knee and right leg pain, chest pain, right arm pain, and head injuries requiring medical treatment. The locations of falls were as follows: 100 in the bathroom (29.94%), 78 around the house (23.35%), 66 in the kitchen (19.76%), 46 in the living room (13.77%), 32 in the bedroom (9.58%), 9 on the stairs (2.70%), and 3 in the garage (0.90%). Most fall locations were included in the assessment tools, except for the following cases: stumbling on one’s own legs (in the kitchen), reaching for clothes on the clothes rack causing loss of balance (in the bedroom), and stepping on the spit of grandchildren (in the living room).

### Comparison between fallers and non-fallers

The characteristics of fallers and non-fallers are shown in Table 2. Compared with non-fallers, fallers tend to be older, reside in one-story elevated houses, have poor visual acuity and have a higher frequency of underlying diseases such hypertension, diabetes, vertigo, urinary continence, and stroke. They do not, however, differ significantly in terms of sex and marital status, balance ability or history of falling in the previous six months. Baseline home fall hazard scores were significantly higher in the fallers than in the non-fallers for all the assessment tools.

**Table 2:** Demographic characteristics of study subjects (n = 450).

Characteristics	Non-fall (n = 327)		Fall (n = 123)		Total (n = 450)		p-value
	n	(%)	n	(%)	n	(%)	
**Age**^¥^ [38, 39]							0.022^a^
Below 70 years	134	(40.98)	36	(29.27)	170	(37.78)	
70 years and older	193	(59.02)	87	(70.73)	280	(62.22)	
Mean (SD) age in years	72.46	(7.99)	73.67	(7.47)	72.74	(7.86)	
**Sex**^†^							0.387
Female	190	(58.10)	77	(62.60)	267	(59.33)	
Male	137	(41.90)	46	(37.40)	183	(40.67)	
**Marital status**^†^							0.265
Single	23	(7.03)	3	(2.44)	26	(5.78)	
Married	222	(67.89)	85	(69.11)	307	(68.22)	
Widowed/Divorced	82	(25.08)	35	(28.45)	117	(26.00)	
**House types**^†^							0.014^a^
One-story non-elevated house	122	(37.31)	36	(29.27)	158	(35.11)	
One-story elevated house	89	(27.22)	51	(41.46)	140	(31.11)	
Two or more stories house	116	(35.47)	36	(29.27)	152	(33.78)	
**Visual acuity**^†^∏							0.024^a^
Good	172	(52.60)	50	(40.65)	222	(49.33)	
Poor	155	(47.40)	73	(59.35)	228	(50.67)	
**Balance ability**^†^Φ							
Poor	53	(16.21)	23	(18.70)	76	(16.89)	
Good	274	(83.79)	100	(81.30)	374	(83.11)	
**History of fall in the previous 6 months**^†^							0.018^a^
No	289	(88.38)	98	(79.67)	387	(86.00)	
Yes	38	(11.62)	25	(20.33)	63	(14.00)	
**Underlying diseases**^†^							
Hypertension	169	(51.68)	70	(56.91)	239	(53.11)	0.322
Diabetes	58	(17.74)	23	(18.70)	81	(18.00)	0.813
Vertigo	5	(1.53)	3	(2.44)	8	(1.78)	0.515
Urinary Incontinence	5	(1.53)	2	(1.63)	7	(1.56)	0.941
Stroke	9	(2.75)	9	(7.32)	18	(4.00)	0.028^a^
**Home fall hazard Score** [Mean (SD)**]**‡							
Modified HOME FAST	6.77	(2.38)	8.49	(2.23)	7.23	(2.46)	< .001 ^a^
Modified HOME FAST-SR	6.21	(2.62)	11.71	(4.23)	7.71	(3.98)	< .001 ^a^
Thai-HFHAT (69 items)	2.88	(2.04)	4.37	(2.26)	3.29	(2.20)	< .001 ^a^
Thai-HFHAT (44 items)	7.79	(2.22)	13.36	(2.87)	9.31	(3.46)	< .001 ^a^
Thai-HFHAT (27 items)	11.02	(3.20)	16.54	(2.76)	12.52	(3.94)	< .001 ^a^

^a^ with statistical significance level of < .05.

† Chi-Square (or Fisher’s exact) test.

‡ Unpair *t*-test.

¥ independent t-test was used to compare difference between the means.

∏ Visual acuity: good = being able to read the numbers from Snellen chart at a distance 6/12 meters more than haft, poor = otherwise [20].

Φ Balance ability: good = being able to stand on one leg for 10 second or longer; poor = otherwise [31].

### Pattern of association between home fall hazard scores and fall probabilities

Fractional polynomial Cox modeling showed that the associations between home fall hazard scores and fall probabilities were nonlinear. When considering both the model efficiency and the ability to appropriately predict fall probability after model estimation, the final models were as follows: two predictor terms with powers of -1 and 3 for the Modified HOMEFAST; two predictor terms with powers of 2 and 2 for the Modified HOMEFAST-SR: one predictor term with a power of 0 for the Thai-HFHAT (69 items); one predictor term with a power of -2 for the Thai-HFHAT (44 items); and two predictor terms with powers of 3 and 3 for the Thai-HFHAT (27 items) (S1 File).

### Model calibration

Fig 2 shows that the predicted fall probabilities increased incrementally—or almost—across the home fall hazard scores for the Thai-HFHAT and its two abbreviated versions. This was not the case for the Modified HOMEFAST and Modified HOMEFAST-SR, where the predicted fall probabilities dropped at the higher scores. All tools slightly underestimated the observed fall probabilities. When inferring from the intercepts and slopes in the calibration plots, the Thai-HFHAT (27 items) was the most perfectly calibrated model (with its intercept not significantly differing from 0 and the lowest magnitude of slope departing from 1), followed by the Thai-HFHAT (44 items) and Modified HOMEFAST-SR (S2 File).

### Discrimination*

Inferring from the original C-statistics in Table 3, the discriminative ability of almost all tools was considered excellent (C-statistics or AUC ≥ 0.8), with the only exception being the Modified HOME FAST, for which the discriminative ability was less than acceptable (C-statistics or AUC < 0.7). The Thai-HFHAT (69 items) had the highest discriminative ability to differentiate between future fallers and non-fallers, followed by the Thai-HFHAT (44 items) and Thai-HFHAT (27 items), and the Modified HOME FAST-SR and Modified HOME FAST had the lowest discriminative ability (Fig 2 and Table 3). The AUC was highest for the Thai-HFHAT (69 items), followed by the Thai-HFHAT (44 items), the Thai-HFHAT (27 items), and the Modified HOME FAST-SR, while the Modified HOME FAST had the lowest and much lower discriminative ability. The relative levels of sensitivity and specificity showed a similar pattern to the C-statistics, which was highest for the Thai-HFHAT (69 items) and lowest for the Modified HOME FAST.

**Table 3:** Predictive validity of five home fall hazard assessment tools.

Parameter	Modified HOME FAST		Modified HOME FAST-SR		Thai-HFHAT (69 items)		Thai-HFHAT (44 items)		Thai-HFHAT (27 items)	
	$\bar{x}$	(95%CI)	$\bar{x}$	(95%CI)	$\bar{x}$	(95%CI)	$\bar{x}$	(95%CI)	$\bar{x}$	(95%CI)
**C-statistics**										
**C**_**org**_	0.67	(0.64, 0.71)	0.80	(0.78, 0.83)	0.88	(0.87, 0.90)	0.88	(0.86, 0.89)	0.84	(0.82, 0.86)
**C**_**boc**_	0.64	-	0.80	-	0.88	-	0.88	-	0.84	-
**%Optimism**	4.91	-	0.07	-	0.08	-	0.08	-	0.14	-
**Cut points**	8	(6.11, 9.89)	9	(7.86, 10.13)	18	(17.21, 18.78)	11	(10.12, 11.88)	15	(13.91, 16.08)
**Sensitivity**	0.68	(0.58, 0.76)	0.75	(0.66, 0.82)	0.94	(0.88, 0.97)	0.89	(0.82, 0.94)	0.81	(0.73, 0.88)
**Specificity**	0.61	(0.56, 0.67)	0.85	(0.81, 0.89)	0.85	(0.81, 0.89)	0.90	(0.86, 0.93)	0.85	(0.80, 0.88)
**PPV**	0.39	(0.35, 0.44)	0.65	(0.58, 0.71)	0.70	(0.64, 0.75)	0.76	(0.70, 0.81)	0.66	(0.60, 0.72)
**NPV**	0.84	(0.80, 0.87)	0.90	(0.87, 0.93)	0.97	(0.95, 0.99)	0.96	(0.93, 0.97)	0.93	(0.89, 0.95)

C_boc_ = Bootstrap optimum-corrected C-statistics, C_org_ = Original C-statistics, CI = Confidence interval, NPV = Negative predictive value, PPV = Positive predictive value, $\bar{x}$ = mean.

Modified HOME FAST = Modified HOME Falls and Accidents Screening Tool, Modified HOME FAST-SR = Modified HOME Falls and Accidents Screening Tool-Self Report, Thai-HFHAT = Thai Home Falls Hazard Assessment Tool.

Further analysis showed that the discriminative ability of the Modified HOME FAST was significantly lower than that of the other tools (Table 4). The discriminative ability of the Thai-HFHAT (69 items) was significantly better than the other tools, with the exception of the Thai-HFHAT (44 items), for which the discriminative abilities were comparable. The discriminative ability of the Thai-HFHAT (27 items) was not significantly better than that of the Modified HOME FAST-SR.

**Table 4:** Comparison of predictive validity among five home fall hazard assessment tools.

Tools	Δ C-statistics† (95%Confidence interval)							
	Modified HOME FAST-SR		Thai-HFHAT (69 items)		Thai-HFHAT (44 items)		Thai-HFHAT (27 items)	
**Modified HOME FAST**	-0.15	(-0.19, -0.11) ^a^	-0.23	(-0.28, -0.18)^a^	-0.22	(-0.27, -0.17)^a^	-0.19	(-0.24, -0.13)^a^
**Modified HOME FAST-SR**			-0.08	(-0.12, -0.04)^a^	-0.07	(-0.11, -0.03)^a^	-0.04	(-0.08, 0.01)
**Thai-HFHAT (69 items)**					0.01	(-0.01, 0.02)	0.04	(0.01, 0.07)^a^
**Thai-HFHAT (44 items)**							0.03	(0.01, 0.06)^a^

Modified HOME FAST = Modified HOME Falls and Accidents Screening Tool, Modified HOME FAST-SR = Modified HOME Falls and Accidents Screening Tool- Self Report, Thai-HFHAT = Thai Home Falls Hazard Assessment Tool.

**†** The differences in the original C-statistical values among the home fall hazard assessment tools. Tools in the first column were the nominators while those in the other columns were the subtractors.

^a^ with statistical significance level of < .05.

### Internal validation

Bootstrap-corrected C-statistics from 1,000 resamplings (Table 3) showed that the discriminative ability of all assessment tools was well reproducible, with negligible magnitude of optimism or overfitting (< 1%) for almost all tools. The exception was that of the Modified HOME FAST, of which the magnitude of optimism was approximately 5%—which was also very low.

The performance of the dichotomized home fall hazard scores is shown in Table 5, which was consistent with the result in Table 3, although it differed in the parameters displayed. The fall hazard ratio was extremely high for those who were classified as the “High” score group according to the Thai-HFHAT (69 items), followed by those of the Thai-HFHAT (44 items) and the Thai-HFHAT (27 items). The contrast in the observed fall probabilities between the “Low” and “High” fall hazard groups was also the most obvious for these three assessment tools. Again, the Modified HOME FAST had the lowest performance, as shown by its lowest hazard ratio and lowest contrast in the observed fall probabilities between the “Low” and “High” fall hazard groups.

**Table 5:** Performance of the dichotomized scores of five home fall hazard assessment tools.

Tools	Adjusted Hazard Ratio†		Observed Fall Probability					
			Low Score‡		High Score‡		High—Low¥	
	HR	(95%CI)	P_obs_	(95%CI)	P_obs_	(95%CI)	ΔP_obs_	(95%CI)
**Modified HOME FAST**								
High versus Low Scores	2.14	(1.48, 3.09)	0.29	(0.24, 0.33)	0.53	(0.49, 0.58)	-0.25	(-0.32, -0.18)
**Modified HOME FAST-SR**								
High versus Low Scores	7.04	(4.37, 11.35)	0.15	(0.12, 0.19)	0.66	(0.62, 0.71)	-0.51	(-0.57, -0.45)
**Thai-HFHAT (69 items)**								
High versus Low Scores	64.44	(30.39, 136.63)	0.03	(0.01, 0.05)	0.67	(0.62, 0.71)	-0.64	(-0.69, -0.58)
**Thai-HFHAT (44 items)**								
High versus Low Scores	33.30	(18.74, 59.15)	0.05	(0.03, 0.08)	0.69	(0.65, 0.73)	-0.64	(-0.69, -0.58)
**Thai-HFHAT (27 items)**								
High versus Low Scores	12.25	(6.96, 21.58)	0.11	(0.08, 0.15)	0.66	(0.62, 0.71)	-0.55	(-0.61, -0.49)

CI = Confidence interval, HR = Hazard ratio, P_obs_ = Observed fall probability.

Modified HOME FAST = Modified HOME Falls and Accidents Screening Tool, Modified HOME FAST-SR = Modified HOME Falls and Accidents Screening Tool-Self Report, Thai-HFHAT = Thai Home Falls Hazard Assessment Tool.

**†** Estimated by Cox proportional hazard model to determine the fall risk of the participants with the baseline home fall hazard scores at the optimal cut-offs or higher compared to those with lower home fall hazard scores and controlled for the potential confounding effect of gender, age, visual acuity, body balance ability, histories of falls and stroke.

**‡** Low and high home fall hazard score groups which were dichotomized basing on the identified optimal cut-off for each home fall hazard assessment tool.

¥ Observed probability of high home fall hazard score group–observed probability of low home fall hazard score group.

While the overall agreement among the dichotomized scoring algorithms of the five assessment tools was weak (Kappa < 0.60), the pairwise agreement among the Thai-HFHAT and its two abbreviated versions was moderate (Kappa of 0.60–0.79) (Table 6). The agreement between the Modified HOMEFAST and the Modified HOMEFAST-SR was weak (Kappa 0.40–0.59), but their agreement with the Thai-HFHAT and its two abbreviated versions was minimal (Kappa 0.21–0.39) or even “none” (Kappa < 0.20) for the agreement between the Modified HOMEFAST and the Thai-HFHAT (27 items).

**Table 6:** Agreement among five home fall hazard assessment tools in classifying subjects into “low” and “high” fall-risk groups.

Tools	Kappa† (95%Confidence interval)							
	Modified HOME FAST-SR		Thai-HFHAT (69 items)		Thai-HFHAT (44 items)		Thai-HFHAT (27 items)	
**Modified HOME FAST**	0.49	0.42, 0.57	0.27	(0.18, 0.36)	0.25	(0.16, 0.34)	0.14	(0.05, 0.23)
**Modified HOME FAST-SR**			0.49	(0.41, 0.58)	0.53	(0.44, 0.61)	0.43	(0.34, 0.52)
**Thai-HFHAT (69 items)**	**Overall Kappa‡**				0.78	(0.72, 0.84)	0.77	(0.70, 0.83)
**Thai-HFHAT (44 items)**	**0.48 (0.42, 0.52)**						0.67	(0.60, 0.75)

Modified HOME FAST = Modified HOME Falls and Accidents Screening Tool, Modified HOME FAST-SR = Modified HOME Falls and Accidents Screening Tool- Self Report, Thai-HFHAT = Thai Home Falls Hazard Assessment Tool.

**†**Cohen/Conger's Kappa (all p < .05).

**‡**Scott/Fleiss' Kappa. (all p < .05).

## Discussion

This study examined the potential applicability of internationally validated home fall hazard assessment tools, namely, the HOME FAST and the HOME FAST-SR, to assess the risk of falls among older Thai adults due to home environmental hazards. The results showed that the applicability in the Thailand context was excellent for the HOME FAST-SR but slightly lower than acceptable for the HOME FAST in terms of discriminative ability to differentiate between future fallers and non-fallers. Further adaptation into local geography, culture, and architectural design resulted in an even better tool in predicting fall risk among older Thai adults. These findings were well reproducible, with negligible or minimal magnitude of optimism.

The predictive validity results were consistent with those reported previously, although the relative magnitude of predictive ability was difficult to directly compare [12, 14, 47–49]. Currently, there are only five published reports on the predictive validity of home fall hazard assessment instruments based on prospective study designs, including two from the US [50, 51], one each from Australia [12] and China [14] and one from Sweden, Germany, and Latvia [49]. However, one study did not specify predictive validity in quantitative terms [13]. Since our reported association of the assessment score with predicted fall probability was nonlinear, although this was not the case for the other studies, estimation of increased fall probability per unit increase in the assessment score was inappropriate. However, we also found that participants with baseline home fall hazard assessment scores of the optimal cutoffs or higher had a higher risk of future falls, with an adjusted HR of 2.14 to 64.44, depending on the type of assessment tool (Table 5). Overall consistency among these studies may result from their sharing of similar general features of the home fall hazard assessment tools. However, the discrepancy in detailed findings was due to the differences in specific features and the scoring procedures of the assessment tools, variations in the definition of fall outcome, the differences in statistical methods used, and the variations in environmental factors according to geography, culture, and architectural design.

Moreover, this study reported sensitivities of 0.68 to 0.94 and specificities of 0.61 to 0.90, which were consistent with the findings of Lai et al., who used the Chinese HOME FAST-SR in predicting falls among 90 and 60 older Chinese male and female adults and showed that the sensitivity and specificity of the instrument at the six-month follow-up were 0.83 and 0.80, respectively [14]. It should be noted that these values were slightly higher than the reported values of our Modified HOME FAST, which were 0.68 and 0.61, respectively, for sensitivity and specificity.

The discriminative abilities of the three versions of Thai-HFHAT were higher than those for the Modified HOME FAST and the Modified HOME FAST-SR, indicating that taking into account the local geographical, cultural, and architectural context is important in the development of home fall hazard assessment instruments specifically for certain populations [28]. Of note was the fact that the two abbreviated versions (44 and 27 items) of the Thai-HFHAT were developed post hoc by a data-driven method and were prone to overfitting [35]. Their discriminative abilities were therefore likely to be optimistic and overestimated. Although the internal validation using the bootstrap optimism-corrected C-statistics showed that the possibility of overfitting and optimism was low, external validation in the independent study population is needed [52]. In terms of the Modified HOME FAST-SR, the finding that its predictive validity was similar to three forms of the Thai-HFHAT assessments also implied that this instrument was well applicable to the international context, as indicated by the fact that a number of investigators had applied this instrument to their home countries [14–16, 53, 54].

Better discriminative abilities of the three versions of the Thai-HFHAT and the Modified HOME FAST-SR than those of the Modified HOME FAST were probably because the former four instruments were evaluated by the elderly, while the last instrument was evaluated by medical personnel only (in this case, a physical therapist). The elderly’s assessment was based on their greater familiarity with their own environment than the medical personnel, resulting in their more accurate identification of home fall hazards than the medical personnel [55]. This was in accord with previous evidence showing that older people consistently scored home fall hazards slightly higher than occupational therapists [13], but other healthcare professionals consistently scored lower than occupational therapists due to the different nature of their knowledge background [55].Considering occupational therapists as experts who had more specialized knowledge of home and environmental hazards, using nonoccupational therapist professionals as the Modified HOME FAST assessors in our study might have resulted in the underestimation of home fall hazards and, thus, its lower discriminative ability. This may be improved by proper training of nonoccupational therapy assessors before home assessment.

Furthermore, since the newly developed Thai-HFHAT may be too long for Thai elderly individuals, two abbreviated versions (with 44 and 27 items) were also developed post hoc based on the two different data analysis methods. These discriminative abilities, together with some aspects of applicability (ease of use and time spent), may indicate that the Thai-HFHAT (44 items) can be suitable for screening fall risks in older Thai adults due to its high sensitivity (0.93) and specificity (0.88) by having fewer question items and requiring only 30 minutes of assessment time, compared with the Modified HOME FAST-SR (87 items), which requires 60 minutes of assessment time. Since the fall incidence reported in this study (27.33%) was comparable to those reported nationally by the Thailand Department of Disease Control (27%) [2], it was therefore expected that it would yield similarly high PPV (0.76) and NPV (0.97) values when applied elsewhere in the country.

The advantages of the present study were as follows. First, its one-year longitudinal design can provide more accurate predictive validity results than the cross-sectional design in most previous studies [42, 43]. Second, since this study had taken into account some variables (such as age, sex, balance, vision, past histories of fall and stroke) in the analysis, the reported discriminative ability was therefore independent of confounding from such variables. Third, since the participants included both genders, the study findings can be applicable to the entire older adult population. Fourth, since most participants were healthy, fall outcomes among them could be truly attributable to home fall hazards rather than the individuals’ internal factors. Some limitations, however, need mentioning. First, although the participants were recruited from diverse areas of the district and covered all main types of houses in Thailand, our use of quota sampling might have resulted in nonrepresentativity in the study population. Second, most of the falls that occurred in the present study were mild, with only 1.20% of injuries requiring medical treatment. Whether our findings will also be applicable for predicting more severe falls is still unconfirmed. Third, while our study results regarding discrimination were highly reproducible, the issue of generalizability has not yet been addressed. Therefore, external validation in another group of participants recruited based on probabilistic sampling to be well represented in the target population is needed to address these three limitations. Last, some fall situations (such as stumbling over one’s legs in the kitchen, reaching for clothes on the clothes rack causing loss of balance in the bedroom, and stepping on the grandchildren’s spit in the living room) were not covered in the assessment tools; thus, further tool improvement is needed.

## Conclusions

This study showed that internationally validated home fall hazard assessment tools such as the HOME-FAST and HOME FAST-SR are well applicable for Thailand, but further adaptation of the tools into a specific local context has yielded even more highly valid tools in predicting fall risk among older Thai adults. When predictive validity and applicability were taken into account, we recommended that the Thai-HFHAT (44 items) be considered the most suitable screening tool due to its high sensitivity and specificity, even with fewer question items and an assessment time of only 30 minutes. These findings were well reproducible. However, due to its post hoc development and some limitations in this study (such as nonprobabilistic sampling), its further external validation in the independent group of well-represented study populations is imperative.

## Supporting information

S1 Table Comparison of Home Falls and Accidents Screening Tool-Self Report (HOME FAST-SR) (87 items) with Thai Home Falls Hazard Assessment Tool (Thai-HFHAT) (69 items). (PDF)

S2 Table Cox proportional hazard model analysis results of each question in Thai Home Falls Hazard Assessment Tool (Thai-HFHAT). (PDF)

S1 File Fractional polynomial method to explore relationship pattern between home fall hazard assessment scores and fall risk among thail elderlies. (PDF)

S2 File Callibration plot of five home fall hazard assessment tool. (PDF)
