# Supplementary material for: Predictive validity of three home fall hazard assessment tools for older adults in Thailand
Source: PLoS One. 2020 Dec 31;15(12):e0244729. doi: 10.1371/journal.pone.0244729 (PMC7774965; doi:10.1371/journal.pone.0244729)
Supplement: S1 Table — (PDF) [file pone.0244729.s001.pdf]

S1 Table. Comparison of Home Falls and Accidents Screening Tool-Self Report (HOME FAST-SR) (87 items) with Thai Home Falls Hazard Assessment Tool (Thai-HFHAT) (69 items).

Item	HOME FAST-SR (87 items)	Thai-HFHAT (69 items)
	**Flooring**	
1	Are the mats secured to the floor?	Similar question but adds "unused cloth", because Thai elderly often use unused cloth as foot towel.<br>"Mats/rugs/unused cloth not fixed to the floor?"<br>- In living room<br>- In kitchen room<br>- In bedroom<br>- In garage
2	Are all carpets free of lumps, holes, tears, loose threads etc.?	No question because most houses in Thailand do not use carpets**.**
3	Do you have any tiled or shiny floors in the kitchen?	Similar question but adds "uneven floor", because the floors of some Thai houses are built with uneven concrete surface.<br>**"**Slippery surface / uneven surface?<br>- In living room<br>- In kitchen room<br>- In bedroom<br>- Stairs (indoor/outdoor)<br>- In garage
4	Do you have any tiled or shiny floors in the bathroom?	Similar question but adds "uneven surface", because some Thai bathroom floors are built with uneven concrete surface.<br>**"**Slippery surface / uneven surface?<br>- In bathroom
5	Do your walkways or hallways have cords lying across them?	Similar question but adds "clutters", because clutters causing to falls for Thai elderly.<br>"Clutters/cords lying across walkway?"<br>- In living room<br>- In kitchen room<br>- In bedroom<br>- Above stairs<br>- In garage
6	Do your walkways or hallways have furniture in them?	Same question<br>"Not enough space for the moment?"<br>- In living room<br>- In kitchen room<br>- In bedroom
7	Do your walkways or hallways have items blocking doorways/doors?	Same as Item 6

Item	HOME FAST-SR<br>(87 items)	Thai-HFHAT<br>(69 items)
8	Do your walkways or hallways have raised thresholds in doorways?	Similar question but adds “different level floors in room”, because some Thai houses floor have different level in room that caused of falling.<br>“Different level floors?”<br>- In living room<br>- In kitchen room<br>- In bedroom<br>- In garage<br>- Around the house
9	Do your walkways or hallways have any other objects or bits and pieces in them?	Same question<br>“Clutters/cords lying across walkway?”<br>- In living room<br>- In kitchen room<br>- In bedroom<br>- Above stairs<br>- In garage<br>- Around the house
	**Furniture**	
10	Does your chair have soft or deep cushions?	No question because soft or deep cushions does not cause falls for Thai elderly.
11	Is your bed the right height for you (not too high or low)?	Same question<br>“The height of bed is not suitable?”
12	Does your bed have a firm mattress?	Same as Item 11
	**Lighting**	
13	At night when you have your room lights on**:** Are your rooms generally bright enough to read a newspaper?	Same question<br>“Lighting is not suitable for activities?”<br>- In living room<br>- In kitchen room<br>- In bathroom<br>- In bedroom<br>- Above stairs (indoor)<br>- In garage<br>- Around the house
14	At night when you have your room lights on**:** Are there any dark shadows across the hallways or floors in your home at night?	Same question<br>“Lighting is not suitable for activities?”
15	Can you turn a light on beside your bed without getting out of bed?	Same question<br>“Unable to turn on the light from the bed?”
16	Do you turn ALWAYS a light on when getting up at night?	Same question<br>“Lighting is not suitable for activities?”
17	Do you use a nightlight that is left on all night to illuminate the route to the bathroom or toilet?	Same as Item 15
18	Do you have a bright light over the back door?	Same as Item 15
19	Do you have a bright light over the front door?	Same as Item 15

Item	HOME FAST-SR (87 items)	Thai-HFHAT (69 items)
20	Are there any shadows across your outdoor paths at night?	Same as Item 15
21	Do you have any excess glare at home during the day?	Same as Item 15
	**Mobility**	
22	Do you use a walking aid to walk around at home?	No question because "use a walking aid to walk around at home" does not cause fall from home hazard.
23	Does it take you several attempts to get up out of your sitting chair?	No question because soft of deep cushions does not cause fall for Thai elderly.
24	When you lower yourself into the chair can you do so without falling back into the chair?	Same as Item 23
25	Does it take you several attempts to get up from the side of the bed?	Same question<br>"The height of bed is not suitable?"
26	When you lower yourself onto the bed can you do so without falling back onto the bed?	Same as Item 25
27		*"Lying on the floor?"*<br>*No question in HOME FAST-SR because some Thai elderly sleep on the floor.*
28	Can you reach items in the kitchen without bending?	Similar question but adds "using a chair or railing to help with handling?"<br>"The cabinet is too low or too high?"<br>- In kitchen room
29	Do you eat in the kitchen?	Same question<br>"Not enough space for the moment?"<br>- In kitchen room
30	Can you carry meals with both hands?	Same as Item 29
31	Do you push meals on a trolley?	No question because Thai eating culture not push meals on a trolley?
	**Bathing**	
32	Is the toilet the right height for you – with or without a raised toilet seat (not too high or low)?	Same question<br>"Toilet is too low or too height?"
33	Do you have to hold onto a sink or other surface to get up from the toilet?	Same as Item 32
34	Do you have a grab rail fitted beside the toilet?	Same question<br>"No grab rail in the bathroom?"
35	Does it take several attempts to get up from the toilet?	Same question<br>"Toilet is too low or too height?"
36	When you lower yourself onto the toilet can you do so without falling back onto the toilet?	Same as Item 35
37	Is your toilet inside the house?	Same question<br>"The bathroom is located outside the house?"

Item	HOME FAST-SR (87 items)	Thai-HFHAT (69 items)
38	Are there any steps/stairs between the toilet and your bedroom?	Similar question but adds “different level floor in bathroom?”. “Different level floors?” In bathroom
39	Is there a long walk between the toilet and your bedroom?	Same question “The bathroom is located outside the house?”
40	Do you get into a bathtub to bathe?	No question because most Thai bathing culture not use bathtub to bathe.
41	Can you safely step over the edge of the bath?	Same as Item 40
42	Can you lower yourself into the bottom of the bath and get up again?	Same as Item 40
43	Do you use non-slip mats or strips in the bathtub?	Same as Item 40
44	Do you have a grab rail beside the bath?	Same as Item 40
45	Do you use a shower over the bath?	No Question because most Thai bathing culture not use a shower over the bath.
46	Do you stand in the bath to shower?	Same as Item 45
47	Do you use a bath board/seat?	Same as Item 45
48	Do you have to hold on to anything to get in and out of the bath when showering?	Same as Item 45
49	Do you have a grab rail?	Same as Item 45
50	Do you use non-slip mats or strips in the bath?	Same as Item 45
51	Do you use a shower recess?	No Question because most Thai bathing culture not use a shower recess.
52	Can you step over the shower hob? (the step or door tracks for the recess)?	Same as Item 51
53	Do you have to hold on to anything to get in and out of the shower recess when showering?	Same as Item 51
54	Do you have a grab rail inside the shower recess?	Same as Item 51
55	Do you use non-slip mats or strips in the shower recess?	Same as Item 51
56	Do you use a shower chair/stool?	Same as Item 51
57		*“The shower area is not separated from the toilet?” This question does not exist in HOME FAST-SR but it was added because local research result showed that separating the bathroom from the toilet was related to reduced fall risk for Thai elderly. [25]*
58		*“Unused cloth not fixed to the floor in bathroom?” This question does not exist in HOME FAST-SR but it was added because Thai elderly often use unused cloth as foot towel.*

Item	HOME FAST-SR (87 items)	Thai-HFHAT (69 items)
59		*"Clutters scattered on the floor in bathroom"*<br>*No question in HOME FAST-SR because Thai bathroom floor is often cluttered with objects.*
60		*"Use squatted-type toilet?"*<br>*This question does not exist in HOME FAST-SR but it was added because many Thai houses use squatted-type toilet.[26]*
61		*"Difficult to handle the accessories in the bathroom?"*<br>*This question does not exist in HOME FAST-SR but it was added because focus group opinion commented that the difficulty in handling the accessories in the bathroom was related to increased fall risk for Thai elderly.*
	**Storage**	
62	Can you reach items in the kitchen without climbing or standing on something?	Same question<br>"The cabinet is too low or too high?"<br>- In kitchen room
	**Steps/Stairs**	
63	Are any of the steps too high to use easily?	Same question<br>"Are the steps of the stairs (indoor/outdoor) too high?"
64	Are any of the treads too narrow for your foot?	Same question<br>"The steps (indoor/outdoor) are smaller than the sole of foot?"
65	Are any of the treads uneven?	Same question<br>"The steps are not the same height (indoor/outdoor)?"
66	Do you get tired/breathless using the steps/stairs?	Same question<br>"Are the steps of the stairs (indoor/outdoor) too high?"
67	Is it difficult to balance on the steps/stairs?	Same question<br>"Are the steps of the stairs (indoor/outdoor) too high?"
68	Are the edges of the steps/stairs easy to see?	Same question<br>"The edge of the steps (indoor/outdoor) is not clear?"
69	Do you have a patterned floor covering on any of the steps/stairs?	Same question<br>"The edge of the steps (indoor/outdoor) is not clear?"
70	Is there enough lighting to see the steps/stairs?	Same question<br>"The light is not suitable for going up and down stairs?"
71	For **ALL indoor** steps/stairs: Are there handrails along the full length of the steps /stairs?	Same question<br>No grab rails (indoor)?
72	For **ALL indoor** steps/stairs: Are the rails easy to put your hand around?	Same question<br>"The rail (indoor) is not strong, unusable?
73	For **ALL indoor** steps/stairs: Are the rails firm and sturdy?	Same question<br>The rail (indoor) is not strong, unusable?
74	For **ALL outdoor** steps/stairs: Are there handrails along the full length of the steps /stairs?	Same question<br>"No grab rails (outdoor)?"
75	For **ALL outdoor** steps/stairs: Are the rails easy to put your hand around?	Same question<br>"The rail (outdoor) is not strong, unusable?

Item	HOME FAST-SR (87 items)	Thai-HFHAT (69 items)
76	For **ALL outdoor** steps/stairs: Are the rails firm and sturdy?	Same question<br>"The rail (outdoor) is not strong, unusable?"
77		*"The stairs are not strong, unusable?"*<br>*This question does not exist in HOME FAST-SR but it was added because local research result showed that the unstable stairs was related to increased fall risk for Thai elderly. [27]*
78		*"No landing?"*<br>*This question does not exist in HOME FAST-SR but it was added because architectural scholar opinion commented that the landing prevented falling for Thai elderly.*
**Around the house**		
79	Is there a landing at the entrance door?	No question because most Thai houses have a landing.
80	Is it easy to lock and unlock the entrance door?	Same question<br>"Using the entrance door is difficult and unsafe?"
81	Can you open your screen door without stepping backwards down any entrance steps?	Same question<br>"Using the entrance door is difficult and unsafe?"
82	Are your outdoor paths cracked?	Same question<br>"The pathway around the house is not in good condition?"
83	Are there any loose pavers in your outdoor paths?	Same as Item 77
84	Do you have gravel walkways at home?	Same as Item 77
85	Are your paths overgrown with plants/grasses/roots?	Same as Item 77
86	Are there overhanging trees over your paths?	Same as Item 77
87	Are there any objects across your paths (e.g. hoses)?	Similar question but adds "shoes placed between the entrance and the outside of the house", because Thai elderly trend to take off their shoes in front of the house.<br>"Clutter/hoses or shoes placed between the entrance and the outside of the house?"
88		*"In case of using a wheelchair, the unappropriated slope is less than 1:12"*<br>*This question does not exist in HOME FAST-SR but it was added because some Thai houses have slope for the elderly who use a wheelchair.*
**Shoes**		
89	Do you go barefoot at home (indoors or outside)?	No question because almost all Thais normally walk barefoot inside house and focus group opinion commented that barefoot does not relate with fall among Thai older adults.
90	Are your shoes firm fitting?	Same question<br>"Wearing inappropriate shoes?"
91	Do your shoes have low heels?	Same as Item 89
92	Do your shoes have a non-slip sole?	Same as Item 89

Item	HOME FAST-SR (87 items)	Thai-HFHAT (69 items)
93	Do they get underfoot when you feed them?	Same question<br>“Having pets inside the house poses a fall risk?”
94	Do you put your pets’ food bowl on the floor when you feed them?	Same as Item 92
95	Do you have to exercise your pets?	Same as Item 92
