# Supplementary material for: Predictive validity of three home fall hazard assessment tools for older adults in Thailand
Source: PLoS One. 2020 Dec 31;15(12):e0244729. doi: 10.1371/journal.pone.0244729 (PMC7774965; doi:10.1371/journal.pone.0244729)
Supplement: S2 Table — (PDF) [file pone.0244729.s002.pdf]

S2 Table. Cox proportional hazard model analysis results of each question in Thai Home Falls Hazard Assessment Tool (Thai-HFHAT).

Questions	Thai-HFHAT (69 Items)		Thai-HFHAT (44 Items)		Thai-HFHAT (27 Items)		
	Adjusted HR (95%CI)	p-value	Adjusted HR (95%CI)	p-value	Adjusted HR (95%CI)	p-value	Frequency (n=450)
**Living room**							
Lighting is not suitable for activities?	2.21 (0.85, 5.77)	0.105	2.18 (0.97, 4.88)	0.058	-	-	19
Slippery surface / uneven surface?	1.23 (0.68, 2.25)	0.492	-	-	1.28 (0.89, 1.86)	0.200	236
Different level floors?	1.60 (0.85, 2.98)	0.142	1.63 (1.00, 2.66)	0.049[a]	1.32 (0.92, 1.89)	0.135	235
Not enough space for the moment?	1.43 (0.82, 2.50)	0.204	1.53 (0.97, 2.41)	0.066	-	-	33
Clutters/cords lying across walkway?	1.78 (1.14, 2.79)	0.011[a]	1.63 (1.17, 2.56)	0.004[a]	1.75 (1.19, 2.57)	0.004[a]	83
Mats/rugs/unused cloth not fixed to the floor?”	1.84 (1.11, 3.05)	0.018[a]	-	-	1.17 (0.84, 1.65	0.357	239
**Kitchen room**							
Lighting is not suitable for activities?	3.03 (1.22, 7.52)	0.017 [a]	-	-	-	-	18
Slippery surface / uneven surface?	0.98 (0.56, 1.70)	0.942	-	-	1.86 (1.15, 3.01)	0.011 [a]	229
Different level floors?	0.70 (0.24, 2.03)	0.512	-	-	0.74 (0.42, 1.31)	0.302	315
Not enough space for the moment?	0.45 (0.19, 1.06)	0.069	0.62 (0.41, 0.94)	0.026[a]	-	-	29
Clutters/cords lying across walkway?	2.52 (1.38, 4.59)	<0.001[a]	2.24 (1.60, 3.13)	<0.001[a]	-	-	51
Mats/rugs/unused cloth not fixed to the floor?”	2.05 (0.94, 4.50)	0.070	2.64 (1.48, 4.73)	0.001[a]	2.16 (1.33, 3.50)	0.002 [a]	335
The cabinet is too low or too high?	4.96 (3.21, 7.66)	<0.001[a]	3.95 (2.61, 5.98)	<0.001[a]	2.67 (1.82, 3.93)	<0.001[a]	140
**Bathroom**							
The bathroom is located outside the house?	3.73 (1.95, 7.13)	<0.001[a]	3.60 (2.26, 5.73)	<0.001[a]	-	-	42
Lighting is not suitable for activities?	2.87 (1.24, 6.64)	0.013[a]	4.50 (2.21, 9.16)	<0.001[a]	-	-	17
Slippery surface?	1.11 (0.65, 1.88)	0.692	-	-	1.06 (0.74, 1.52)	0.751	131
Different level floors?	3.63 (2.06, 6.43)	<0.001 [a]	3.62 (3.22, 5.88)	<0.001 [a]	1.94 (1.27, 2.96)	0.002 [a]	320
Clutters scattered on the floor in bathroom?	0.10 (0.03, 0.41)	0.001 [a]	0.13 (0.04, 0.42)	0.001 [a]	-	-	19
Unused cloth not fixed to the floor in bathroom?	2.41 (1.49, 3.90)	<0.001[a]	1.84 (1.26, 2.70)	0.002 [a]	0.93 (0.70, 1.23)	0.619	321

Questions	Thai-HFHAT (69 Items)		Thai-HFHAT (44 Items)		Thai-HFHAT (27 Items)		
	**Adjusted HR (95%CI)**	**p-value**	**Adjusted HR (95%CI)**	**p-value**	**Adjusted HR (95%CI)**	**p-value**	**Frequency (n=450)**
The shower area is not safe?	2.79 (1.48, 5.25)	0.001[a]	3.01 (1.89, 4.81)	<0.001 [a]	-	-	19
The shower area is not separated from the toilet?	2.50 (1.18, 5.29)	0.017 [a]	1.59 (1.06, 2.38)	0.025 [a]	2.14 (1.15, 3.97)	0.016 [a]	336
Unused a bath seat?	1.38 (0.86, 2.23)	0.183	1.35 (0.96, 1.90)	0.086	0.87 (0.61, 1.26)	0.472	310
No grab rail in the bathroom?	1.06 (0.60, 1.86)	0.852	-	-	1.35 (0.75, 2.44)	0.322	317
Use squatted-type toilet?	3.80 (2.06, 7.01)	0.001 [a]	3.36 (1.95, 5.79)	<0.001 [a]	3.10 (2.01, 4.76)	<0.001 [a]	185
Toilet is too low or too height?	2.62 (1.46, 4.73)	0.001 [a]	2.03 (1.34, 2.08)	0.001	-	-	72
Difficult to handle the accessories in the bathroom?	4.14 (0.28, 6.15)	0.302	-	-	-	-	4
**Bedroom**							
Lighting is not suitable for activities?	2.16 (0.90, 5.20)	0.085	2.21 (1.01, 4.84)	0.047 [a]	-	-	33
Unable to turn on the light from the bed?	2.32 (1.26, 4.28)	0.007 [a]	1.96 (1.30, 2.95)	0.010 [a]	2.08 (1.44, 3.02)	<0.001 [a]	331
Slippery surface / uneven surface?	1.34 (0.74, 2.42)	0.327	1.57 (1.08, 2.28)	0.019 [a]	1.35 (0.96, 1.92)	0.086	197
Different level floors?	1.77 (1.00, 3.14)	0.049 [a]	2.32 (1.52, 3.55)	<0.001 [a]	-	-	84
Not enough space for the moment?	2.21 (0.96, 5.11)	0.061	3.21 (2.04, 5.06)	<0.001 [a]	-	-	32
Clutters/cords lying across walkway?	1.71 (1.11, 2.66)	0.016	1.71 (1.20, 2.44)	0.003 [a]	-	-	70
Mats/rugs/unused cloth not fixed to the floor?”	0.66 (0.37, 1.16)	0.151	0.65 (0.46, 0.94)	0.020 [a]	1.00 (0.68, 1.38)	0.864	225
Lying on the floor?	0.60 (0.37, 0.96)	0.034 [a]	-	-	-	-	186
The height of bed is not suitable?	1.31 (0.77, 2.25)	0.322	-	-	-	-	122
The cabinet is too low or too high?	0.08 (0.02, 0.32)	<0.001 [a]	0.21 (0.08, 0.55)	0.001 [a]	-	-	12
**Stairs**							
The light is not suitable for going up and down stairs?	0.42 (0.03, 6.02)	0.526 [a]	0.13 (0.04, 0.41)	0.001 [a]	-	-	7
Slippery surface?	1.07 (0.35, 3.24)	0.91	-	-	-	-	38
Clutters/cords lying across walkway?	2.71 (1.24, 5.90)	0.012 [a]	4.64 (2.43, 8.86)	<0.001 [a]	-	-	29
Are the steps of the stairs (indoor) too high?”	2.78 (1.14, 6.75)	0.024 [a]	3.76 (1.98, 7.13)	<0.001 [a]	-	-	44
The edge of the steps (indoor) is not clear?	0.24 (0.08, 0.70)	0.009 [a]	-	-	0.68 (0.26, 1.73)	0.414	56
The steps are not the same height (indoor)?	0.32 (0.07, 1.45)	0.140	0.34 (0.14, 0.87)	0.023 [a]	-	-	25

Questions	Thai-HFHAT (69 Items)		Thai-HFHAT (44 Items)		Thai-HFHAT (27 Items)		
	Adjusted HR (95%CI)	p-value	Adjusted HR (95%CI)	p-value	Adjusted HR (95%CI)	p-value	Frequency (n=450)
The steps (indoor) are smaller than the sole of foot?	2.66 (0.82, 8.61)	0.103	2.04 (1.16, 3.57)	0.013 [a]	-	-	30
No grab rails (indoor)?	3.25 (1.17, 9.03)	0.024 [a]	2.23 (1.39, 3.57)	0.001 [a]	1.33 (0.59, 2.99)	0.484	75
The stair and rail (indoor) are not strong, unusable?	4.61 (0.89, 23.79)	0.068	5.75 (2.19, 15.12)	<0.001 [a]	-	-	20
No landing?	1.09 (0.36, 3.31)	0.881	-	-	-	-	59
**Garage**							
Lighting is not suitable for activities?	14.39 (1.36,15.26)	0.027 [a]	4.44 (1.13, 17.51)	0.033	-	-	2
Slippery surface?	2.33 (1.00, 5.46)	0.050 [a]	2.21 (1.26, 3.89)	0.006 [a]	-	-	29
Uneven surface?	1.86 (1.08, 3.22)	0.026 [a]	-	-	-	-	71
Different level floors?	2.31 (1.44, 3.70)	<0.001 [a]	1.99 (1.30, 3.03)	0.001 [a]	1.91 (1.34, 2.72)	<0.001 [a]	117
Clutters/cords lying across walkway?	0.47 (0.20, 1.12)	0.009 [a]	0.53 (0.30, 0.96)	0.036	1.31 (0.91, 1.89)	0.143	60
Mats/rugs/unused cloth not fixed to the floor?	0.99 (0.39, 2.51)	0.987	-	-	-	-	27
The cabinet is too low or too high?	1.47 (0.58, 3.77)	0.419	2.87 (1.36, 6.08)	0.006 [a]	-	-	16
**Around the house**							
Lighting is not suitable for activities?	1.59e -20	-	-	-	-	-	12
Using the entrance door is difficult and unsafe?	0.93 (0.33, 2.59)	0.889	-	-	-	-	27
Different level floors?	0.44 (0.19, 1.04)	0.062	-	-	0.52 (0.27, 0.97)	0.042 [a]	384
Clutters/cords lying across walkway?	1.27 (0.73, 2.20)	0.391	-	-	1.19 (0.86, 1.65)	0.290	138
The pathway around the house is not in good condition?	1.69 (1.09, 2.62)	0.019 [a]	1.73 (1.16, 2.59)	0.008 [a]	1.41 (1.03, 1.94)	0.035 [a]	134
Slippery surface?	0.91 (3.54, 33.64)	<0.001 [a]	9.59 (4.20, 21.89)	<0.001 [a]	-	-	26
Are the steps of the stairs (outdoor) too high?	0.01 (0.001, 0.19)	0.001 [a]	0.05 (0.01 0.28)	0.001 [a]	-	-	7
The edge of the steps (outdoor) is not clear?	0.51 (0.07, 3.74)	0.511	-	-	-	-	42
The steps are not the same height (outdoor)?	1.27 (0.18, 8.90)	0.809	-	-	-	-	6

Questions	Thai-HFHAT (69 Items)		Thai-HFHAT (44 Items)		Thai-HFHAT (27 Items)		
	**Adjusted HR (95%CI)**	**p-value**	**Adjusted HR (95%CI)**	**p-value**	**Adjusted HR (95%CI)**	**p-value**	**Frequency (n=450)**
The steps (outdoor) are smaller than the sole of foot?	136.45 (2.91, 6395.99)	0.012 [a]	15.87 (3.57, 70.45)	0.001 [a]	-	-	2
No grab rails (outdoor)?	1.85 (1.00, 3.41)	0.048	-	-	-	-	65
The stair and rail (outdoor) are not strong, unusable?	0.10 (0.003, 2.91)	0.178	0.10 (0.02, 0.43)	<0.001 [a]	-	-	24
No landing?	2.59 (0.61, 11.01)	0.196	-	-	-	-	25
In case of using a wheelchair, the unappropriated slope is less than 1:12	3.77^{e-18}	-	2.74e -18	-	-	-	2
**Shoes**							
Wearing inappropriate shoes?	1.22 (0.78, 1.91)	0.390	-	-	1.22 (0.88, 1.68)	0.234	136
**Pets**							
Having pets inside the house poses a fall risk?	0.82 (0.52, 1.28)	0.371	-	-	1.33 (0.96, 1.83)	0.082	267
