# Supplementary material for: Predictive validity of three home fall hazard assessment tools for older adults in Thailand
Source: PLoS One. 2020 Dec 31;15(12):e0244729. doi: 10.1371/journal.pone.0244729 (PMC7774965; doi:10.1371/journal.pone.0244729)
Supplement: S1 File — (PDF) [file pone.0244729.s003.pdf]

S1 FILE

FRACTIONAL POLYNOMIAL METHOD
TO EXPLORE RELATIONSHIP PATTERN
BETWEEN
HOME FALL HAZARD ASSESSMENT SCORES
AND FALL RISK AMONG THAI ELDERLIES

Modified HOMEFAST

Exploration

```
. fp <ModifiedHOMEFASTTotal>,replace : stcox <ModifiedHOMEFASTTotal>,  /*
>   */ efron nolog noshow cluster (id) strata ( Seq )
(fitting 44 models)
(....10%....20%....30%....40%....50%....60%....70%....80%....90%....100%)

Fractional polynomial comparisons:
```

Modif~TTotal	df	Deviance	Dev. dif.	P(*)	Powers
omitted	0	1788.742	48.322	--	
linear	1	1759.817	19.397	--	1
m = 1	2	1741.741	1.321	--	-2
m = 2	4	1740.420	0.000	--	-1 3

```
(*) deviance difference test not valid with cluster(id)

Stratified Cox regr. -- Efron method for ties

No. of subjects      =          450          Number of obs    =        560
No. of failures      =          217
Time at risk         =       127909
                                             Wald chi2(2)     =      22.00
Log pseudolikelihood =   -870.20999          Prob > chi2      =     0.0000

                                 (Std. Err. adjusted for 450 clusters in id)
```

_t	Haz. Ratio	Robust Std. Err.	z	P>|z|	[95% Conf.	Interval]
Modi~TTotal_1	3.14e-07	1.04e-06	-4.50	0.000	4.63e-10	.0002123
ModifiedHO~_2	.9996636	.0001552	-2.17	0.030	.9993594	.9999678

Stratified by Seq

Final Selected Model

```
. stcox ModifiedHOMEFASTTotal_1 ModifiedHOMEFASTTotal_2 , /*
>   */ efron nolog noshow cluster (id) strata ( Seq )

Stratified Cox regr. -- Efron method for ties

No. of subjects      =          450          Number of obs    =         560
No. of failures      =          217
Time at risk         =       127909
                                              Wald chi2(2)     =       22.00
Log pseudolikelihood =   -870.20999          Prob > chi2      =      0.0000

                              (Std. Err. adjusted for 450 clusters in id)
```

_t	Haz. Ratio	Robust Std. Err.	z	P>|z|	[95% Conf.	Interval]
ModifiedHOMEFASTTotal_1	3.14e-07	1.04e-06	-4.50	0.000	4.63e-10	.0002123
ModifiedHOMEFASTTotal_2	.9996636	.0001552	-2.17	0.030	.9993594	.9999678

Stratified by Seq

Modified HOMEFAST-SR

Exploration

```
. fp <ModifiedHOMEFASTSRTotal>,replace : stcox <ModifiedHOMEFASTSRTotal>,  /*
>   */ efron nolog noshow cluster (id) strata ( Seq )
(fitting 44 models)
(....10%....20%....30%....40%....50%....60%....70%....80%....90%....100%)

Fractional polynomial comparisons:
---------------------------------------------------------------------
Modif~RTotal |   df    Deviance    Dev. dif.   P(*)    Powers
-------------+-------------------------------------------------------
     omitted |    0    1788.742     186.690     --
      linear |    1    1634.668      32.617     --     1
       m = 1 |    2    1612.073      10.022     --     -.5
       m = 2 |    4    1602.051       0.000     --     2 2
---------------------------------------------------------------------
(*) deviance difference test not valid with cluster(id)

Stratified Cox regr. -- Efron method for ties

No. of subjects      =          450           Number of obs    =         560
No. of failures      =          217
Time at risk         =       127909
                                              Wald chi2(2)     =       81.57
Log pseudolikelihood =    -801.0257           Prob > chi2      =      0.0000

                                (Std. Err. adjusted for 450 clusters in id)
-----------------------------------------------------------------------------------------
                           |               Robust
                        _t | Haz. Ratio   Std. Err.      z    P>|z|     [95% Conf. Interval]
---------------------------+-------------------------------------------------------------
ModifiedHOMEFASTSRTotal_1 |   1.103752   .0209815     5.19   0.000     1.063385     1.14565
ModifiedHOMEFASTSRTotal_2 |   .9708036   .0060172    -4.78   0.000     .9590815    .9826691
-----------------------------------------------------------------------------------------
                                                             Stratified by Seq
```

Final Selected Model

```
. stcox ModifiedHOMEFASTSRTotal_1 ModifiedHOMEFASTSRTotal_2 , /*
>   */ efron nolog noshow cluster (id) strata ( Seq )

Stratified Cox regr. -- Efron method for ties

No. of subjects      =          450            Number of obs    =        560
No. of failures      =          217
Time at risk         =       127909
                                               Wald chi2(2)     =      81.57
Log pseudolikelihood =    -801.0257            Prob > chi2      =     0.0000

                                 (Std. Err. adjusted for 450 clusters in id)
------------------------------------------------------------------------------
                          |               Robust
                       _t | Haz. Ratio   Std. Err.      z    P>|z|     [95% Conf. Interval]
--------------------------+---------------------------------------------------------------
ModifiedHOMEFASTSRTotal_1 |   1.103752   .0209815     5.19   0.000     1.063385     1.14565
ModifiedHOMEFASTSRTotal_2 |   .9708036   .0060172    -4.78   0.000     .9590815    .9826691
------------------------------------------------------------------------------------------
                                                             Stratified by Seq
```

THAI-HFHAT (69 items)

Exploration

```
. fp <Total_HFHAT>,replace : stcox <Total_HFHAT>,  /*
>   */ efron nolog noshow cluster (id) strata ( Seq )
(fitting 44 models)
(....10%....20%....30%....40%....50%....60%....70%....80%....90%....100%)

Fractional polynomial comparisons:

Total_HFHAT    df     Deviance    Dev. dif.   P(*)    Powers

    omitted     0     1788.742     418.264      --
     linear     1     1596.911     226.433      --    1
      m = 1     2     1419.681      49.204      --    -2
      m = 2     4     1370.477       0.000      --    0 .5

(*) deviance difference test not valid with cluster(id)

Stratified Cox regr. -- Efron method for ties

No. of subjects      =          450          Number of obs    =         560
No. of failures      =          217
Time at risk         =       127909
                                             Wald chi2(2)     =      128.44
Log pseudolikelihood =    -685.2387          Prob > chi2      =      0.0000

                                (Std. Err. adjusted for 450 clusters in id)

                          Robust
          _t   Haz. Ratio   Std. Err.       z    P>|z|     [95% Conf. Interval]

Total_HFHAT_1    6.20e+25    6.04e+26    6.10   0.000      3.18e+17    1.21e+34
Total_HFHAT_2    2.37e-10    9.60e-10   -5.48   0.000      8.60e-14    6.55e-07

                                                                 Stratified by Seq
```

Final Selected Model

```
. fp <Total_HFHAT>, fp(0) replace : stcox <Total_HFHAT>,  /*
>   */ efron nolog noshow cluster (id) strata ( Seq )
-> stcox Total_HFHAT_1, efron nolog noshow cluster (id) strata ( Seq )

Stratified Cox regr. -- Efron method for ties

No. of subjects      =          450         Number of obs    =        560
No. of failures      =          217
Time at risk         =       127909
                                            Wald chi2(1)     =      56.52
Log pseudolikelihood =   -757.76368         Prob > chi2      =     0.0000

                                 (Std. Err. adjusted for 450 clusters in id)
```

_t	Haz. Ratio	Robust Std. Err.	z	P>|z|	[95% Conf.	Interval]
Total_HFHAT_1	111.206	69.69099	7.52	0.000	32.5607	379.8065

Stratified by Seq

THAI-HFHAT (44 items)

Exploration

```
. fp <Total44>,replace : stcox <Total44>,  /*
>   */ efron nolog noshow cluster (id) strata ( Seq )
(fitting 44 models)
(....10%....20%....30%....40%....50%....60%....70%....80%....90%....100%)

Fractional polynomial comparisons:
```

Total44	df	Deviance	Dev. dif.	P(*)	Powers
omitted	0	1788.742	389.161	--	
linear	1	1584.419	184.838	--	1
m = 1	2	1431.257	31.677	--	-2
m = 2	4	1399.581	0.000	--	.5 .5

```
(*) deviance difference test not valid with cluster(id)

Stratified Cox regr. -- Efron method for ties

No. of subjects      =          450          Number of obs    =         560
No. of failures      =          217
Time at risk         =       127909
                                             Wald chi2(2)     =      121.05
Log pseudolikelihood =   -699.79047          Prob > chi2      =      0.0000

                                  (Std. Err. adjusted for 450 clusters in id)
```

_t	Haz. Ratio	Robust Std. Err.	z	P>|z|	[95% Conf.	Interval]
Total44_1	2.71e+23	1.96e+24	7.45	0.000	1.85e+17	3.97e+29
Total44_2	.0000173	.0000264	-7.17	0.000	8.62e-07	.0003458

Stratified by Seq

Final Selected Model

```
. fp <Total44>, fp(-2) replace : stcox <Total44>,  /*
>   */ efron nolog noshow cluster (id) strata ( Seq )
-> stcox Total44_1, efron nolog noshow cluster (id) strata ( Seq )

Stratified Cox regr. -- Efron method for ties

No. of subjects      =          450          Number of obs    =        560
No. of failures      =          217
Time at risk         =       127909
                                             Wald chi2(1)     =     102.34
Log pseudolikelihood =   -715.62873          Prob > chi2      =     0.0000

                                  (Std. Err. adjusted for 450 clusters in id)
```

_t	Haz. Ratio	Robust Std. Err.	z	P>|z|	[95% Conf.	Interval]
Total44_1	1.1e-198	4.7e-197	-10.12	0.000	4.6e-237	2.4e-160

Stratified by Seq

THAI-HFHAT (27 items)

Exploration

```
. fp <Total27>,replace : stcox <Total27>,  /*
>   */ efron nolog noshow cluster (id) strata ( Seq )
(fitting 44 models)
(....10%....20%....30%....40%....50%....60%....70%....80%....90%....100%)
```

Fractional polynomial comparisons:

Total27	df	Deviance	Dev. dif.	P(*)	Powers
omitted	0	1788.742	254.657	--	
linear	1	1565.820	31.736	--	1
m = 1	2	1551.200	17.115	--	-.5
m = 2	4	1534.084	0.000	--	3 3

(*) deviance difference test not valid with cluster(id)

Stratified Cox regr. -- Efron method for ties

```
No. of subjects      =          450          Number of obs    =        560
No. of failures      =          217
Time at risk         =       127909
                                             Wald chi2(2)     =      87.56
Log pseudolikelihood =   -767.04209          Prob > chi2      =     0.0000
```

(Std. Err. adjusted for 450 clusters in id)

_t	Haz. Ratio	Robust Std. Err.	z	P>|z|	[95% Conf.	Interval]
Total27_1	1.007536	.0012846	5.89	0.000	1.005022	1.010057
Total27_2	.9977771	.0003944	-5.63	0.000	.9970044	.9985505

Stratified by Seq

Final Selected Model

```
. stcox Total27_1 Total27_2 , /*
>   */ efron nolog noshow cluster (id) strata ( Seq )

Stratified Cox regr. -- Efron method for ties

No. of subjects      =          450          Number of obs    =         560
No. of failures      =          217
Time at risk         =       127909
                                             Wald chi2(2)     =       87.56
Log pseudolikelihood =   -767.04209          Prob > chi2      =      0.0000

                                 (Std. Err. adjusted for 450 clusters in id)
```

_t	Haz. Ratio	Robust Std. Err.	z	P>|z|	[95% Conf.	Interval]
Total27_1	1.007536	.0012846	5.89	0.000	1.005022	1.010057
Total27_2	.9977771	.0003944	-5.63	0.000	.9970044	.9985505

Stratified by Seq
