# Supplementary material for: Predictive validity of three home fall hazard assessment tools for older adults in Thailand
Source: PLoS One. 2020 Dec 31;15(12):e0244729. doi: 10.1371/journal.pone.0244729 (PMC7774965; doi:10.1371/journal.pone.0244729)
Supplement: S2 File — (PDF) [file pone.0244729.s004.pdf]

S2 FILE

CALIBRATION PLOT

OF

FIVE HOME FALL HAZARD ASSESSMENT TOOLS

#	Calibration Plot	Intercept and Slope

1 **Modified HOMEFAST**

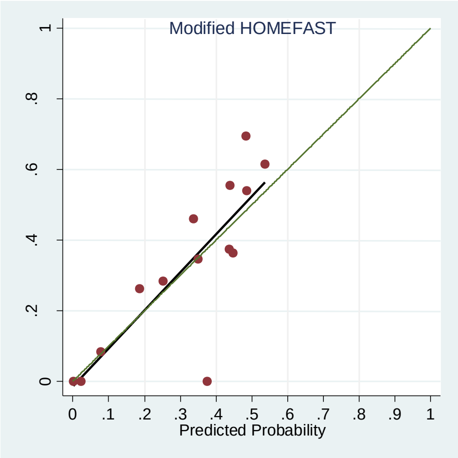


```
. regress x_hf_o x_hf_p [fweight = no_hf]

      Source |       SS           df       MS      Number of obs   =       784
-------------+----------------------------------   F(1, 782)       =   4123.96
       Model |   23.621637         1   23.621637   Prob > F        =    0.0000
    Residual |  4.47921432       782  .005727896   R-squared       =    0.8406
-------------+----------------------------------   Adj R-squared   =    0.8404
       Total |  28.1008514       783  .035888699   Root MSE        =    .07568

------------------------------------------------------------------------------
      x_hf_o |      Coef.   Std. Err.      t    P>|t|     [95% Conf. Interval]
-------------+----------------------------------------------------------------
      x_hf_p |   1.271341   .0197972    64.22   0.000     1.232479    1.310203
       _cons |  -.0221923   .0074846    -2.97   0.003    -.0368847   -.0074999
------------------------------------------------------------------------------
```

2 **Modified HOMEFAST-SR**

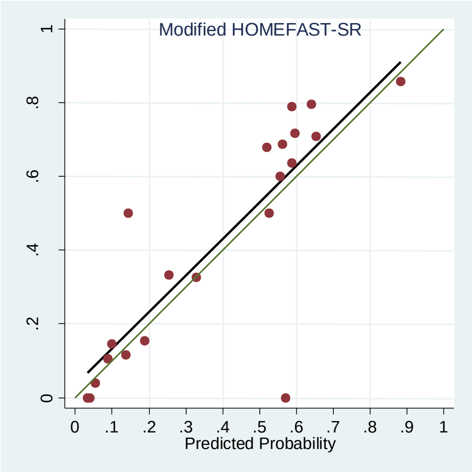


```
. regress x_hfsr_o x_hfsr_p [fweight = no_hfsr]

      Source |       SS           df       MS      Number of obs   =       549
-------------+----------------------------------   F(1, 547)       =   5363.35
       Model |  36.5383029         1  36.5383029   Prob > F        =    0.0000
    Residual |  3.72648665       547   .00681259   R-squared       =    0.9075
-------------+----------------------------------   Adj R-squared   =    0.9073
       Total |  40.2647895       548  .073475893   Root MSE        =    .08254

------------------------------------------------------------------------------
    x_hfsr_o |      Coef.   Std. Err.      t    P>|t|     [95% Conf. Interval]
-------------+----------------------------------------------------------------
    x_hfsr_p |   1.177108    .016073    73.23   0.000     1.145535     1.20868
       _cons |  -.0120189   .0059932    -2.01   0.045    -.0237915   -.0002463
------------------------------------------------------------------------------
```

#	Calibration Plot	Intercept and Slope
3	**THAI-HFHAT (69 items)**	. regress x_thfhat_o x_thfhatr_p0 [fweight = no_thfhat]

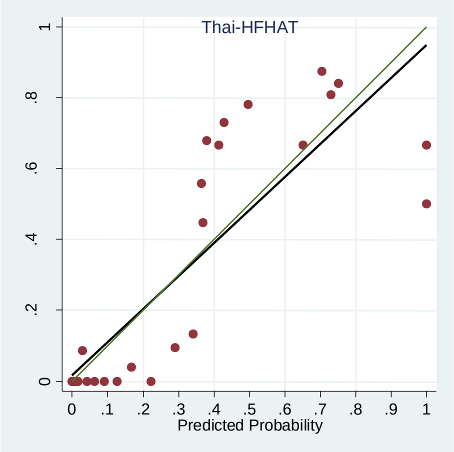


```
. regress x_thfhat_o x_thfhatr_p0 [fweight = no_thfhat]

      Source |       SS           df       MS      Number of obs   =       560
-------------+----------------------------------   F(1, 558)       =   1788.37
       Model |  48.7065828         1  48.7065828   Prob > F        =    0.0000
    Residual |  15.1971917       558  .027235111   R-squared       =    0.7622
-------------+----------------------------------   Adj R-squared   =    0.7618
       Total |  63.9037745       559  .114318022   Root MSE        =    .16503

------------------------------------------------------------------------------
  x_thfhat_o |      Coef.   Std. Err.      t    P>|t|     [95% Conf. Interval]
-------------+----------------------------------------------------------------
x_thfhatr_p0 |   1.332661   .0315131    42.29   0.000     1.270762     1.39456
       _cons |  -.0979922   .0119233    -8.22   0.000    -.1214123   -.0745721
------------------------------------------------------------------------------
```

#	Calibration Plot	Intercept and Slope
4	**THAI-HFHAT (44 items)**	. regress x_thfhat44_o x_thfhatr44_p2 [fweight = no_thfhat44]

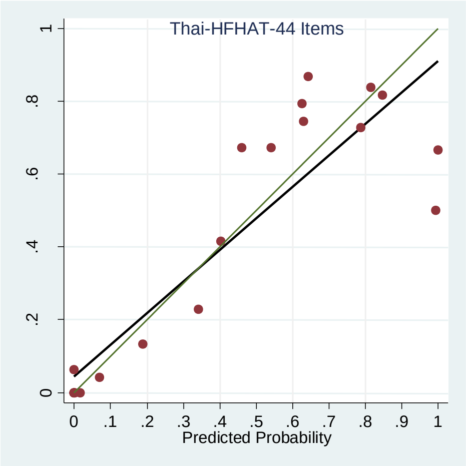


```
. regress x_thfhat44_o x_thfhatr44_p2 [fweight = no_thfhat44]

      Source |       SS           df       MS      Number of obs   =       560
-------------+----------------------------------   F(1, 558)       =   6852.85
       Model |  54.3955931         1  54.3955931   Prob > F        =    0.0000
    Residual |  4.42921462       558  .007937661   R-squared       =    0.9247
-------------+----------------------------------   Adj R-squared   =    0.9246
       Total |  58.8248077       559  .105232214   Root MSE        =    .08909

------------------------------------------------------------------------------
x_thfhat44_o |      Coef.   Std. Err.      t    P>|t|     [95% Conf. Interval]
-------------+----------------------------------------------------------------
x_thfhatr44~2 |   1.114715   .0134657    82.78   0.000     1.088265    1.141165
       _cons |  -.0160536   .0055174    -2.91   0.004     -.026891   -.0052162
------------------------------------------------------------------------------
```

#	Calibration Plot	Intercept and Slope
5	**THAI-HFHAT** (**27 items**) 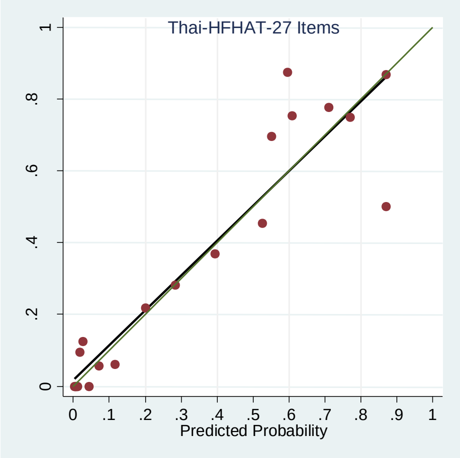	. regress x_thfhat27_o x_thfhatr27_p [fweight = no_thfhat27]

Source	SS	df	MS
Model	44.6829223	1	44.6829223
Residual	3.34764441	558	.005999363
Total	48.0305667	559	.085922302

Number of obs	=	560
F(1, 558)	=	7447.94
Prob > F	=	0.0000
R-squared	=	0.9303
Adj R-squared	=	0.9302
Root MSE	=	.07746

x_thfhat27_o	Coef.	Std. Err.	t	P>|t|	[95% Conf.	Interval]
x_thfhatr27_p	1.099859	.0127444	86.30	0.000	1.074826	1.124892
_cons	-.0015316	.0050767	-0.30	0.763	-.0115033	.00844
